# Human Cytomegalovirus Immunoglobulin G Response and Pulmonary Tuberculosis in Adolescents: A Case-Control Study

**DOI:** 10.1093/ofid/ofad487

**Published:** 2023-09-29

**Authors:** Jeremi Swanepoel, Gert van Zyl, Anneke C Hesseling, Sarah M Johnson, David A J Moore, James A Seddon

**Affiliations:** Desmond Tutu TB Centre, Department of Paediatrics and Child Health, Faculty of Medicine and Health Sciences, Stellenbosch University, Cape Town, South Africa; Department of Clinical Research, London School of Hygiene and Tropical Medicine, London, United Kingdom; Division of Medical Virology, Department of Pathology, Faculty of Medicine and Health Sciences, Stellenbosch University and National Health Laboratory Service, Tygerberg Academic Hospital, Cape Town, South Africa; Desmond Tutu TB Centre, Department of Paediatrics and Child Health, Faculty of Medicine and Health Sciences, Stellenbosch University, Cape Town, South Africa; Desmond Tutu TB Centre, Department of Paediatrics and Child Health, Faculty of Medicine and Health Sciences, Stellenbosch University, Cape Town, South Africa; Department of Infectious Diseases, Imperial College London, London, United Kingdom; TB Centre, London School of Hygiene and Tropical Medicine, London, United Kingdom; Desmond Tutu TB Centre, Department of Paediatrics and Child Health, Faculty of Medicine and Health Sciences, Stellenbosch University, Cape Town, South Africa; Department of Infectious Diseases, Imperial College London, London, United Kingdom

**Keywords:** adolescents, cytomegalovirus, immunology, tuberculosis

## Abstract

**Background:**

Emerging evidence suggests a link between infection with herpes viruses, particularly human cytomegalovirus (HCMV) and Epstein-Barr virus (EBV), and progression to tuberculosis disease.

**Methods:**

An unmatched case-control study was conducted among adolescents aged 10–19 years enrolled in an observational study (Teen TB) between November 2020 and November 2021, in Cape Town, South Africa. Fifty individuals with pulmonary tuberculosis and 51 healthy tuberculosis-exposed individuals without tuberculosis were included. Demographics and clinical data were obtained, and serum samples collected at enrolment were tested for HCMV immunoglobulin G (IgG) and EBV nuclear antigen (EBNA) IgG using 2 automated enzyme immunoassays. Odds ratios were estimated using unconditional logistic regression.

**Results:**

The median age of 101 participants was 15 years (interquartile range, 13–17 years); 55 (54%) were female. All participants were HCMV IgG seropositive, and 95% were EBNA IgG seropositive. Individuals with tuberculosis had higher HCMV IgG titers than healthy controls (*P* = .04). Individuals with upper-tertile HCMV IgG titers had 3.67 times greater odds of pulmonary tuberculosis than those with IgG titers in the lower tertile (95% confidence interval, 1.05–12.84; *P* = .04). There was a trend for increasing odds of pulmonary tuberculosis with increasing titers of HCMV IgG (*P* = .04). In contrast, there was no association between tuberculosis and higher EBNA IgG values.

**Conclusions:**

There is a high prevalence of sensitization to HCMV and EBV among adolescents in this high-tuberculosis-burden setting. Higher HCMV IgG titers were associated with pulmonary tuberculosis in adolescents.

Human cytomegalovirus (HCMV) is a ubiquitous double-stranded DNA herpesvirus that causes asymptomatic and symptomatic infection in immunocompetent hosts. There is evidence suggesting an association between tuberculosis and HCMV [1]. There are similarities in the host immunological response to both HCMV and *Mycobacterium tuberculosis,* and the epidemiological overlap between the 2 diseases is generating considerable interest but is not fully understood. Areas of overlap include humans being the primary host, similar risk factors, and both organisms being able to remain latent in host cells for years before reactivation [2]. Furthermore, the peak of adolescent tuberculosis is mirrored by increasing HCMV seroprevalence, and commentators have suggested that there may be overlapping immune manipulation mechanisms between HCMV and *M tuberculosis* [2–4].

To date, much of the work on *M. tuberculosis* and HCMV coinfection has been done in adults. Two studies that analyzed serum samples from a predominantly adult rural Ugandan cohort found that tuberculosis was associated with higher HCMV immunoglobulin G (IgG) titers [5] and that, after adjusting for human immunodeficiency virus (HIV) infection, higher HCMV IgG was also associated with lower levels of certain antimycobacterial antibodies [6]. Similarly, in one study from Russia, children and adolescents with tuberculosis disease progression were found to have significantly higher HCMV IgG titers than nonprogressors [7]. In South African infants, an HCMV-specific interferon (IFN) γ response was associated with increased risk of developing tuberculosis [8]. These findings suggest that immunological alterations following HCMV infection could play a role in progression from *M tuberculosis* infection to tuberculosis disease.

Epstein-Barr virus (EBV), another ubiquitous double-stranded DNA virus, is a known driver of T-cell activation with a high prevalence in many sub-Saharan African countries [9]. In young children, an EBV-specific CD8^+^ T-cell response following primary infection has been found to contain the virus without subsequent T-cell overexpansion [10]. However, infection in adolescence is characterized by extensive expansion of activated EBV-specific CD8^+^ T cells [11] and is associated with a strong type 1 IFN response and unrestrained inflammation. The immunological effects of EBV infection in adolescence and overlapping age distribution with tuberculosis, as is the case for HCMV, point toward a possible relationship between the 2 pathogens and warrant further investigation.

A systematic review and meta-analysis of epidemiological studies have provided further evidence for an association between HCMV infection and risk of tuberculosis disease [12]. However, much remains unknown about the mechanisms that are responsible for the increased susceptibility to tuberculosis. Furthermore, no studies have yet examined this association in adolescents specifically. Further efforts to unravel the *M tuberculosis*–HCMV interaction may help to identify adolescents who are at greater risk of developing tuberculosis disease. We investigated the association between HCMV and EBV-specific IgG responses and the risk/odds of pulmonary tuberculosis disease among adolescents in a high-tuberculosis-burden setting.

## METHODS

### Study Design and Setting

An unmatched case-control study was carried out to evaluate the association between humoral responses to HCMV and EBV and the odds of tuberculosis disease in adolescents. The study used baseline clinical data and serum samples collected from participants of a cohort study (Teen TB) in Cape Town, situated in the Western Cape province of South Africa, with a high tuberculosis incidence [13, 14].

Teen TB was a prospective observational cohort study that aimed to provide a better understanding of the biology, morbidity, and social contexts of adolescent tuberculosis and how these interact. One of the study objectives was to explore how pubertal hormones and viral coinfections (notably HCMV) influence the immune response to *M tuberculosis.* The study recruited adolescents (aged 10 to <20 years) with routinely diagnosed microbiologically confirmed drug-susceptible or new multidrug-resistant pulmonary tuberculosis disease (with or without HIV coinfection) and clinically well tuberculosis-exposed adolescents.

Adolescents with tuberculosis disease, who were within the first 14 days of diagnosis, were recruited from local clinics in Tygerberg, Mitchell's Plain, and Khayelitsha, within the City of Cape Town Metropole. Tuberculosis-exposed controls from the same subdistricts, who were exposed in their household to a case of infectious pulmonary tuberculosis within the last 6 months, were recruited from clinics where case patients were recruited. Household contacts of enrolled adolescents with tuberculosis were also included. Adolescents with tuberculosis disease were excluded if extrapulmonary tuberculosis, without evidence of pulmonary tuberculosis, was present. Tuberculosis case patients and tuberculosis-exposed controls were excluded if they were clinically unstable or required intensive care treatment, if they were pregnant or breastfeeding, if they were known to have diabetes mellitus, or if they declined HIV testing with no recent (within <12 months) HIV test result available.

Teen TB was a broader study with multiple aims and separate analyses. In the current study, we have focused primarily on HCMV and EBV serological data in adolescents with or without tuberculosis disease. As case patients and controls in Teen TB were unmatched, regression modelling was also used to address confounding and adjust for imbalances between the study groups in our study. A detailed description of the Teen TB study methods and procedures can be found elsewhere [15].

### Study Participants and Sampling

The study population comprised all adolescents 10 to <20 years of age who were recruited into Teen TB between November 2020 and November 2021 and had available baseline demographic, clinical and laboratory data. This included 50 adolescents from Teen TB with microbiologically confirmed pulmonary tuberculosis and available serum samples. One stored serum sample, collected at recruitment and within 14 days of tuberculosis diagnosis, was identified and retrieved for each individual with pulmonary tuberculosis (case patient). For tuberculosis-exposed healthy controls, who remained free of pulmonary tuberculosis disease since their enrollment until November 2021, a stored serum sample collected at recruitment was also retrieved.

### Laboratory Analyses

Previous HCMV infection was defined by the presence of IgG antibodies against HCMV in stored baseline serum, determined using a commercial serology test. Previous EBV infection was defined by the presence of IgG antibodies against EBV nuclear antigen (EBNA), determined using a commercial serology test. Baseline serum was stored as aliquots in 200-μL tubes at −80°C until use. Once thawed, all serum samples were tested with the automated semiquantitative enzyme-linked immunofluorescent bioMérieux VIDAS HCMV IgG and VIDAS EBNA IgG assays to detect IgG antibodies against HCMV and EBNA, respectively. The IgG antibody titers in seropositive samples were also determined. Both bioMérieux VIDAS assays have been used for several years with reliable performance in many reference laboratories [16, 17]. The default unit for the VIDAS HCMV IgG assay is arbitrary units per milliliter; values ≥6 or <4 AU/mL were defined as positive and negative, respectively, according to the manufacturer's instructions. For the VIDAS EBNA IgG assay, the default unit is test value (TV); values ≥0.21 or ≤0.09 TV were defined as positive and negative, respectively. All serological testing was performed by National Health Laboratory Service staff at Tygerberg Academic Hospital, according to the manufacturer's protocols.

### Statistical Analysis

Data were analyzed using Stata software (version 17; StataCorp). Study population characteristics were described as frequencies (percentages), means and standard deviations, or medians and interquartile ranges (IQRs), as appropriate.

Pearson correlation was used to investigate associations between continuous measurements of HCMV and EBNA IgG. Nonnormally distributed virus-specific IgG titers were log-transformed, and box plots were generated to present the distribution of log-transformed virus-specific IgG titers for case and control groups. A Mann-Whitney *U* test was used to assess whether the distribution of HCMV and EBNA IgG values differed between case patients and controls. Samples that were HCMV and EBNA IgG seropositive were categorized into tertiles according to the IgG titer, and the association between herpesvirus IgG tertiles and pulmonary tuberculosis disease was investigated using unconditional logistic regression. Age (continuous), sex, and HIV status were included in the multivariable model as potential confounders, and a likelihood ratio test for association as well as trend was performed.

### Patient Consent Statement

Ethical approval was provided by the Stellenbosch University Health Research Ethics Committee (reference N19/10/148). Parents/legal guardians (for those <18 years old) or participants (aged 18 to <20 years) provided written informed consent for use of clinical data and biological samples, with assent provided by those <18 years. The London School of Hygiene Tropical Medicine also provided ethical approval for this study (reference 26000).

## RESULTS

### Characteristics of Study Participants

A total of 101 adolescents were included. The median age (IQR) was 15 (13–17) years, 55 participants (54%) were female, and 6 (5.9%) were HIV positive. Of those who were HIV positive, 3 were not on antiretroviral therapy (HIV infection had been newly diagnosed in 2, and 1 had defaulted treatment). Among those with pulmonary tuberculosis, 40 had drug-susceptible tuberculosis, and 10 had multidrug-resistant tuberculosis. Among healthy tuberculosis-exposed controls, 69% (35/51) had evidence of *M tuberculosis* infection (positive IFN-γ release assay test result). All participants were HCMV IgG seropositive, and 95% (96 of 101) were EBNA IgG seropositive (Table 1). HCMV IgG titers were not associated with EBNA IgG titers (*r* [97] = −0.01; *P* = .92).

**Table 1.** Baseline Sociodemographic and Clinical Characteristics for the Study Population

Characteristic	Study Participants, No. (%)^a^	
	Case Patients (n = 50)	Controls (n = 51)
Age, median (IQR), y	17.1 (15.2–18.7)	13.9 (11.6–15.5)
Sex		
Male	18 (36.0)	28 (54.9)
Female	32 (64.0)	23 (45.1)
Ethnicity		
Black African	27 (54.0)	31 (60.8)
Mixed race	23 (46.0)	20 (39.2)
Housing type^b^		
Formal	42 (84.0)	39 (76.5)
Informal	8 (16.0)	12 (23.5)
Household size		
≤5 Members	28 (56.0)	28 (54.9)
≥6 Members	22 (44.0)	23 (45.1)
Cooking fuel		
Electricity or gas	49 (98.0)	49 (96.1)
Paraffin or coal	1 (2.0)	2 (3.9)
Water source		
Inside tap	42 (84.0)	42 (82.3)
Outside tap	8 (16.0)	9 (17.7)
Toilet location		
Inside house	42 (84.0)	37 (72.5)
Outside house	8 (16.0)	14 (27.5)
Missing	0 (0.0)	0 (0.0)
Primary caregiver		
Parent	43 (86.0)	47 (92.2)
Nonparent^c^	7 (14.0)	4 (7.8)
Anyone in household employed		
No	10 (20.0)	6 (11.8)
Yes	40 (80.0)	45 (88.2)
Household smoking exposure^d^		
No	19 (38.0)	13 (25.5)
Yes	31 (62.0)	38 (74.5)
Current smoker		
No	32 (64.0)	45 (88.2)
Yes	18 (36.0)	6 (11.8)
Tuberculosis signs and symptoms		
Cough	42 (84.0)	2 (3.9)
Wheeze	20 (40.0)	0 (0.0)
Fever	7 (14.0)	0 (0.0)
Lack of appetite	21 (42.0)	0 (0.0)
Weight loss	39 (78.0)	1 (2.0)
Night sweats	29 (58.0)	0 (0.0)
Lymphadenopathy	4 (8.0)	1 (2.0)
Chronic lung disease signs^e^		
No	49 (98.0)	51 (100.0)
Yes	1 (2.0)	0 (0.0)
BCG vaccine scar		
No	0 (0.0)	1 (2.0)
Yes	49 (98.0)	49 (96.0)
Missing	1 (2.0)	1 (2.0)
Previous tuberculosis disease		
No	41 (82.0)	51 (100.0)
Yes	9 (18.0)	0 (0.0)
HIV status		
Negative	45 (90.0)	50 (98.0)
Positive	5 (10.0)	1 (2.0)
HCMV IgG serostatus		
Negative	0 (0.0)	0 (0.0)
Positive	50 (100.0)	51 (100.0)
EBNA IgG serostatus		
Negative	2 (4.0)	3 (5.9)
Positive	48 (96.0)	48 (94.1)

Abbreviations: HIV, human immunodeficiency virus; IgG, immunoglobulin G; IQR, interquartile range.

^a^Data represent no. (%) of participants unless otherwise specified.

^b^Formal was defined as a brick house; informal, as a prefabricated wooden house or shanty house.

^c^Nonparents included grandmothers or grandfathers, other family members, or community members.

^d^Household smoking exposure was defined as exposure to secondhand tobacco smoke in the household.

^e^Signs of chronic lung disease included chest deformity, clubbing, coarse crackles, and pulmonary hypertension.

### HCMV IgG Response and Association With Pulmonary Tuberculosis

**Figure 1.**
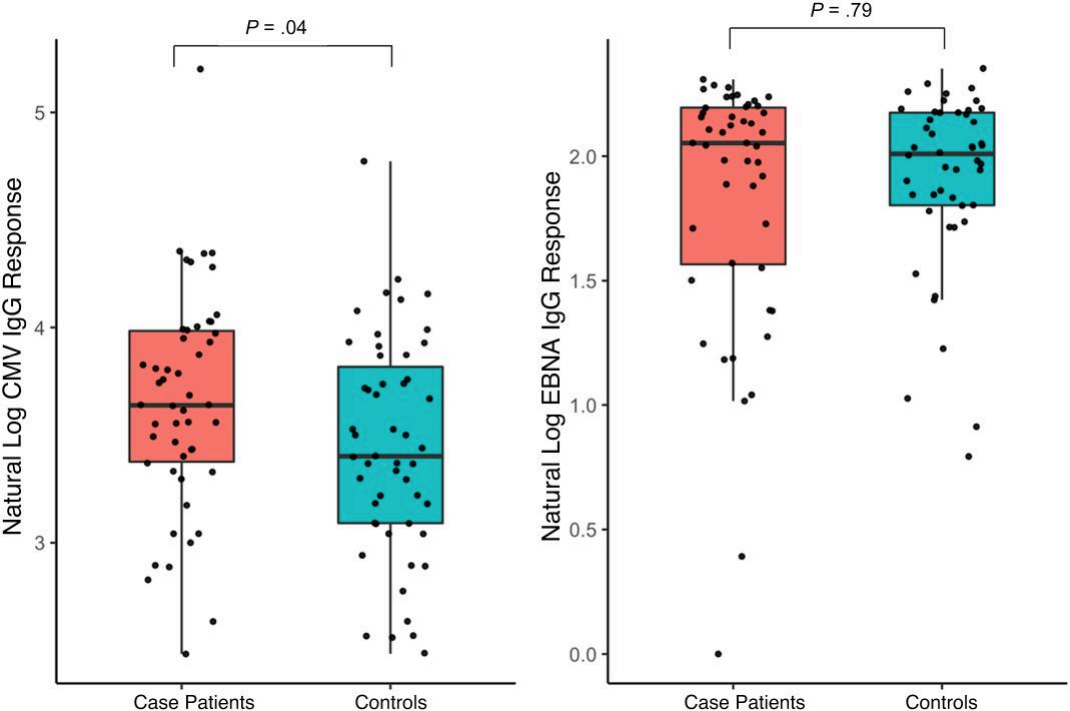
Log-transformed human cytomegalovirus (HCMV) and Epstein-Barr virus nuclear antigen (EBNA) immunoglobulin G (IgG) response values for Teen TB case and control groups, with accompanying *P* values (Mann-Whitney *U* test).

Adolescents with tuberculosis had a higher median (IQR) HCMV IgG value than those without tuberculosis: 38 (29–54) versus 30 (22–48) AU/mL, respectively (Figure 1; *P* = .04). After adjustment for age, sex, and HIV status, those with middle-tertile HCMV IgG titers had 3.18 times increased odds of pulmonary tuberculosis disease compared with those with lower-tertile IgG titers (95% confidence interval [CI], .89–11.32; *P* = .07) (Table 2). Upper-tertile HCMV IgG values were associated with 3.67 times greater odds of pulmonary tuberculosis disease compared with lower-tertile IgG titers (95% CI, 1.05–12.84; *P* = .04). There was a trend toward increasing odds of pulmonary tuberculosis disease with increasing HCMV IgG tertile (*P* = .04).

**Table 2.** Odds of Pulmonary Tuberculosis by Virus-Specific Immunoglobulin G Titer

Virus-Specific IgG Titers	No. of Serum Samples	Adjusted OR (95% CI)^a^	*P* Value for Trend^b^
**HCMV IgG titers by tertile**			
Lower (12.00–28.00 AU/mL)	33	1.0 (Reference)	.04
Middle (29.00–43.00 AU/mL)	34	3.18 (.89–11.32)	
Upper (44.00–182.00 AU/mL)	34	3.67 (1.05–12.84)	
**EBNA IgG titers by tertile**			
Lower (1.00–6.49 TV)	32	1.0 (Reference)	.62
Middle (6.50–8.53 TV)	32	1.15 (.34–3.82)	
Upper (8.54–10.53 TV)	32	1.39 (.40–4.82)	

Abbreviations: AU, arbitrary units; CI, confidence interval; EBNA, Epstein-Barr virus nuclear antigen; HCMV, human cytomegalovirus; IgG, immunoglobulin G; OR, odds ratio; TV, test value.

^a^Middle and upper tertiles are compared with the lower tertile of IgG titers in an unconditional logistic regression model. ORs are adjusted for age, sex, and human immunodeficiency virus status.

^b^ *P* value from a likelihood ratio test for trend.

### EBNA IgG Response and Association With Pulmonary Tuberculosis

Adolescents with tuberculosis who were EBNA IgG seropositive had a median (IQR) IgG value of 7.79 (4.77–9.01) TV, compared with 7.46 (6.07–8.82) TV for seropositive controls (Figure 1; *P* = .79). There was no statistically significant difference in the odds of having pulmonary tuberculosis between those with middle-tertile EBNA IgG titers and those with lower-tertile IgG titers (odds ratio, 1.15 [95% CI, .34–3.82]; *P* = .83) (Table 2). There was no increase in the odds of pulmonary tuberculosis in those with EBNA IgG values in the upper tertile versus the lower tertile (odds ratio, 1.39 [95% CI, .40–4.82]; *P* = .60).

## DISCUSSION

In this case-control study, our findings suggest that the magnitude of HCMV IgG response is associated with an increased odds of pulmonary tuberculosis disease in adolescents, in a dose-response manner. Despite evidence for coprevalence of HCMV and EBV infections, the same association with tuberculosis disease risk was not seen with increasing EBNA IgG titers.

In Africa, most children are infected with HCMV early in infancy irrespective of HIV exposure or infection [18–20]. During adolescence, the risk of HCMV infection and exposure load increases further owing to changes in personal behaviors and exposures that place adolescents at risk of acquiring infections. Multiple possible mechanisms have been described, by which HCMV infection may augment *M tuberculosis* infection and increase tuberculosis disease risk. Both *M tuberculosis* and HCMV infect the same cells, and HCMV has been found to encode a variety of viral proteins that can interact with, and potentially disrupt, *M tuberculosis* containment and increase tuberculosis disease risk by interfering with the host's local protective immune responses [21–23].

Imbalances in systemic cytokine responses and production by macrophages and T cells (CD8^+^ and γδ cells) caused by HCMV infection have also been recognized [24]. Furthermore, initial antiviral responses by host cells against HCMV infection favor the production of type 1 IFNs, which have been found to detrimentally affect the course of *M tuberculosis* infection, especially when high levels of IFN are produced [25]. In this study we evaluated only the association between magnitude of IgG response and odds of disease. HCMV-specific IgG titers serve as a measure of cumulative HCMV exposure, and it is likely that HCMV reactivation or (re)infection events may be the driver for increased HCMV IgG titers in the studied population. It is postulated that alterations to the wider immunological environment and the inflammation caused by either (re)infection or fluctuation between periods of latency and active HCMV disease could provide conditions conducive to progression from *M tuberculosis* infection to tuberculosis disease.

Our results show a pattern similar to those from the few available studies that have examined the association between HCMV IgG response and tuberculosis disease. Stockdale and colleagues [26] found that increased serum HCMV IgG titers in a predominantly adult Ugandan cohort (mean age, 34 years) were associated with an increased odds of progression to symptomatic tuberculosis. The same association with tuberculosis disease risk was not seen with other herpesviruses, such as EBV and herpes simplex virus 1 and 2. In that study, adults with high HCMV IgG titers were found to have 3.4 times greater odds of having pulmonary tuberculosis disease than those with low HCMV IgG titers.

Martinez and colleagues [27] conducted a prospective, population-based, birth cohort study in a periurban setting outside of Cape Town, South Africa, and found that children who acquired an HCMV infection early in life were at increased risk of tuberculosis disease during their childhood years and that those with high HCMV loads were at greatest risk of tuberculosis disease. In that study, HCMV infection was not associated with tuberculin skin test positivity among South African infants. These findings, in combination with our findings, further support the existence of a consistent biological gradient (dose-response relationship) whereby subsequent risk of tuberculosis disease is modified by greater HCMV exposure load. Our study is the first to examine this relationship in a purely adolescent cohort and extends these earlier findings from adult and birth cohorts.

Our study has some limitations. It involved a relatively small group of adolescents and only evaluated IgG responses to HCMV and EBNA. In addition, HCMV IgG antibodies were measured from serum samples collected at baseline only; IgG titers may change over time within individuals. Moreover, our study only investigated HCMV IgG responses in a tuberculosis-endemic settings, where exposure to HCMV and other herpesviruses is ubiquitous, and it is unclear whether reactivation events or repeated infections are driving the observed effect. Given that two-thirds of the controls from the Teen TB study were from the same household as an enrolled tuberculosis case, factors such as socioeconomic status, lifestyle, diet and household crowding were controlled for to some extent, but the risk of residual confounding still remains. We were, however, able to adjust for important confounding factors that have been shown to be associated with increased HCMV IgG seropositivity, such as increasing age, female sex, and HIV positivity. Finally, the grouping into HCMV IgG tertiles was based on the IgG titers found in our adolescent population and may not be generalizable to other populations with different HCMV and EBV infection dynamics.

To assist with characterizing the effects of HCMV on *M tuberculosis* and vice versa, future immunological studies evaluating T-cell activation/responses and biological pathways are needed. Improved characterization of the HCMV, tuberculosis, and HIV coinfection status of individuals in clinical cohorts will aid in determining whether HCMV is a predictor for mortality or morbidity in tuberculosis. Numerous candidate HCMV vaccines are currently in development, but target groups include mainly pregnant women and transplant recipients [28]. Given that there is already early evidence of reduction in HCMV infections among seronegative individuals in phase II HCMV vaccine studies [28], it might be possible to use vaccines to avert progression of *M tuberculosis* infection to tuberculosis disease in the future.

The current study contributes to growing evidence that the magnitude of IgG response to HCMV is associated with tuberculosis disease. Our results suggest that more urgency is needed in investigating strategies to reduce HCMV exposure and IgG antibody titers among HCMV-infected adolescents.

## Notes

***Acknowledgments.*** We thank the study participants, their families, and the research staff at the Desmond Tutu TB Centre for their dedication and support. We also thank Shannon Wilson from the Division of Medical Virology at Stellenbosch University and National Health Laboratory Service at Tygerberg Hospital for preparing samples and assisting with the serological assays.

***Author contributions.*** D. A. J. M. and J. A. S. conceptualized the study and supervised the work. J. A. S. provided the necessary data and assisted with developing the study methodology. G. v. Z. coordinated the virological testing and provided input on appropriate assays to use. A. C. H. and S. M. J. provided additional methodological and/or clinical advice. J. S. assisted with laboratory assays, analyzed data, and wrote the manuscript. All authors read, edited, and approved the final manuscript.

***Financial support.*** This work was supported by the UK Medical Research Council (MRC) and the UK Department for International Development (DFID) (joint funding of Clinician Scientist Fellowship MR/R007942/1 to J. A. S., under the MRC/DFID Concordat agreement) and by the Wellcome Trust (grant RSRO_P67869 to S. M. J.). Funding for the serological assays and testing was provided by the Johnson & Johnson COVID-19 Research and Travel Fund.
